# Health-related quality of life among people with rheumatic and musculoskeletal diseases in Cyprus: a cross-sectional study of disease burden and time since diagnosis

**DOI:** 10.1186/s41927-026-00617-z

**Published:** 2026-01-20

**Authors:** Nikoleta Nikolaou, Victor Hadjiroussos, Christiana Nicolaou, Michalis Michaelides, Andrie G. Panayiotou, Nicos Middleton, rECORD project team

**Affiliations:** 1 Department of Nursing, School of Health Sciences, Cyprus University of Technology, Limassol, Cyprus; 2 Cyprus Rheumatology Society, Nicosia, Cyprus; 3 Department Rehabilitation Sciences, School of Health Sciences, Cyprus International Institute for Environmental and Public Health, Cyprus University of Technology, Limassol, Cyprus; 4 Partnership Epidemiological Community Study of Rheumatic Diseases (rECORD) in Association with Cyprus Rheumatology Society and Cyprus League of People with Rheumatic Diseases, Nicosia, Cyprus

**Keywords:** Rheumatic diseases, Musculoskeletal diseases, Quality of life, Cross-Sectional studies, Time factors, Cyprus, Disease progression, Surveys and questionnaires, Chronic disease, Health status

## Abstract

**Background:**

Rheumatic and musculoskeletal diseases (RMDs) profoundly affect health-related quality of life (HRQoL), imposing a significant distinct burden on physical and mental well-being. Given the high prevalence and long-term disability associated with RMDs, understanding their impact is essential for informing public health priorities. This study aims to delineate the multifaceted impact of RMDs on HRQoL across diverse disease categories and examine how the impact on HRQoL varies by diagnosis and time since diagnosis.

**Methods:**

This cross-sectional study (July 2023 - July 2024) employed a mixed sampling approach, combining a stratified sample of participants from clinical settings with an open call through online channels to reach a broader population of individuals with RMD in Cyprus. Physical (PCS) and Mental Component Scores (MCS) of the SF-12 questionnaire were used to assess HRQoL. Demographic and clinical variables, including age, gender, marital status, smoking status, and time since diagnosis were recorded. Multivariable regression analyses were used to explore differences in HRQoL scores by disease group and time since diagnosis, adjusting for demographic characteristics.

**Results:**

The study enrolled 789 participants (mean age 55.4 ± 13.2 years, 76.6% female). Among participants, 57.4% reported low PCS (< 40) and 38.7% reported low MCS (< 40). Fibromyalgia demonstrated the most pronounced HRQoL impairments, with significantly lower scores, compared to inflammatory diseases, for both physical (B = − 11.45; 95% CI: − 15.06, − 7.83) as well as mental components of HRQoL (B = − 12.31; 95% CI: − 16.18, − 8.4). Notable reductions in PCS and MCS were also recorded among patients with rheumatoid arthritis (RA) and systemic lupus erythematosus (SLE) reflecting the systemic and psychological burden of these conditions. Marked HRQoL reductions were recorded among participants in early disease stages (0–2 years), while those with a longer time since diagnosis (2–10 years) demonstrated relative improvements.

**Conclusion:**

RMDs significantly impact HRQoL, with substantial variability across disease categories and time since diagnosis. While the cross-sectional nature of the study does not capture trajectories across stages of disease progression, the significant and differential by disease category impairment in HRQoL at the early stages emphasize the need for disease-specific, multidisciplinary care approaches and highlight the critical importance of early diagnosis and optimized management to mitigate the long-term impact of RMD.

**Supplementary Information:**

The online version contains supplementary material available at 10.1186/s41927-026-00617-z.

## Introduction

Rheumatic and musculoskeletal diseases (RMDs) are a broad group of chronic conditions affecting millions of people worldwide. They directly impact the physical health of individuals through chronic pain, disability and systemic inflammation, which are hallmark characteristics of these conditions [1]. However, mental health is also significantly affected in people with RMD, with an increased risk of depression, anxiety and fatigue [2]. These complex and multifaceted effects increase disease burden and negatively impact patients’ well-being [3]. Many studies in the past have highlighted the significant deterioration in health-related quality of life (HRQoL) in people with RMDs compared to the general population; however, there is considerable heterogeneity in the degree of impairment across specific disease categories [4–6].

RMDs represent a major public health problem due to their high prevalence, their contribution to long-term disability, and their substantial impact on workforce participation and healthcare utilization [1]. Their diverse clinical manifestations, ranging from localized joint disorders to systemic inflammatory conditions further complicate disease management. The heterogenous and multifaceted nature of RMDs highlights the need for tailored and integrated approaches to disease-related care [7]. In addition to physical symptoms, the psychological burden associated with these conditions further complicates disease management, but it is not consistently addressed in routine care [8, 9].

Disease duration also emerges as an important determinant of HRQoL of people with RMDs. In the early stages, the intense inflammatory response leads to acute physical damage and challenges in adapting to a new situation. As the diseases progress into middle and advanced stages greater variability is observed. The effect of disease duration on HRQoL is reflected in factors such as treatment effectiveness, the development of coping mechanisms, and the accumulation of irreversible physical damage over time [10]. Although disease duration is recognized as an important determinant of HRQoL, the complex interplay between disease characteristics, disease stage, and overall impact on HRQoL remains incompletely understood across different populations and healthcare settings [11].

Providing integrated care and universal access to health services is a major challenge for all health systems. The healthcare system in Cyprus has recently undergone a major reform, unifying the public and private sectors, to ensure universal access. However, national policies, clinical guidelines, referral protocols and care pathways are still evolving, and in the case of RMDs, are largely nonexistent. Thus, monitoring the HRQoL of people with RMDs provides a valuable snapshot of patient well-being and may offer complementary insights into aspects of chronic disease care and management. This is particularly relevant in settings undergoing healthcare system transitions, where variations in care coordination, continuity, and access to specialist and integrated services may shape patient experiences. Given the significant burden of disability and the socioeconomic consequences associated with RMDs, especially among working-age populations, such data can help contextualize patient-reported outcomes within broader efforts to improve prevention, early diagnosis, and disease management strategies.

This study aims to evaluate HRQoL outcomes associated with RMDs in the Cypriot population, focusing on disease category and disease duration. The research was conducted in a setting with limited epidemiological data on the prevalence of RMDs and little evidence regarding unmet needs and clinical practices, particularly with respect to primary care screening practices and referrals, time to diagnosis, and care management. Furthermore, this is occurring in the context of the recent transition to the new general healthcare system (GeSY) which is still characterized by the uncertain readiness of primary care to identify RMD and specialists who, anecdotally, are overwhelmed by the excessive numbers of patients diagnosed with RMDs as well as new referrals from family doctors, a lot of which are integrative. Understanding the physical and mental health impacts of RMDs within this evolving healthcare system underscores the importance of developing disease-and stage-specific interventions and integrating patient-reported outcomes into policy and practice to ensure effective monitoring and long-term care planning.

## Methodology

### Study aim and design

The primary aim of this study was to estimate the burden of RMDs on physical and mental HRQoL. The objectives were to explore the degree of differences in HRQoL based on specific disease characteristics, including diagnosis and disease duration. As this was the first study in Cyprus addressing the HRQoL of people with RMDs, rather than focusing on the trajectory of disease-specific impact on HRQoL, it was deemed more important to capture a broad and diverse sample of people with RMD, across diverse diseases categories, different stages since diagnosis and representative of different care settings. Thus, a cross-sectional design was chosen to provide a comprehensive snapshot of HRQoL variability across more disease categories and time since diagnosis. Data collection took place during the period July 2023 to July 2024.

### Sample and sampling

A mixed sampling approach was employed combining a stratified sample of participants from various clinical settings with an open call through online channels to reach a broader population of people with RMDs in Cyprus. Stratified sampling ensured the inclusion of people with RMDs from diverse clinical settings. Recruitment at clinics involved visits to rheumatologists’ offices across Cyprus, three days per site, four hours per visit. Given the lack of formal records on the number of people attending at each site and the case-mix, it was deemed important to apply a fixed visit timeframe across all participating sites.

To identify clinical sites, the research team contacted the Cyprus Rheumatology Society, who provided a list of all registered Rheumatology Specialists with their contact details. All were invited to participate in the study and visits were to be arranged to their practice according to a standard schedule. A total of 18 sites participated, including both rheumatologists’ offices in the community- and hospital-based rheumatologist clinics, operating within the Cyprus State Health Services Organization. This corresponds to a participation rate of approximately 70%.

In rheumatologists’ offices in the community, patients typically receive care from a single rheumatologist operating independently with a more personalized approach but potentially with limited resources. In contrast, hospital-based clinics are those which operate within the public healthcare system, where multiple healthcare professionals provide care in an outpatient setting typically managing a larger volume of patients and offering multidisciplinary support.

To complement recruitment from clinical sites and in order to expand the number of patients with RMD, a parallel online sample collection was conducted, collecting comparable data on demographic and clinical characteristics as well as HRQoL. The online survey was conducted in collaboration with the Cyprus League Agents Rheumatic Diseases (https://rheumatism.org.cy/), which promoted the online questionnaires through social media channels, email lists and their newsletter. For participants recruited through online channels, diagnoses were self-reported and clinical verification by a rheumatologist was not feasible. In contrast, for participants recruited from rheumatology clinics, diagnoses were confirmed through routine clinical assessment.

Eligible participants were adults (≥ 18 years) with a self-reported physician diagnosis of any RMD, regardless of time since diagnosis. Additional inclusion criteria were fluency in Greek and/or English and ability to provide informed consent. Individuals with comorbidities were not excluded, reflecting the real-world clinical complexity of RMDs. Participants were excluded if they were younger than 18 years, unable to provide informed consent, or had insufficient proficiency in Greek/English.

A single primary diagnosis was used for analytical purposes. Approximately 12% of participants reported more than one diagnosis. For participants recruited from rheumatology clinics, the primary diagnosis was determined by the treating rheumatologist as the clinically dominant condition guiding management at the time of assessment. For participants recruited through online channels, where clinical verification was not feasible, the primary diagnosis was self-designated by the participant. No case was double-counted across disease categories, and no distinction between primary and secondary diagnosis was made.

### Data collection

Data collection across clinical sites was carried out by two trained investigators to ensure a consistent approach in data collection and adherence to ethical standards. Questionnaires were administered in person during patients’ waiting time at clinic visits. Questionnaires were completed independently by the participants. Individuals with physical limitations (e.g. impaired hand function or visual difficulties) were provided with assistance as needed in completing the questionnaires. Participation in the online survey was voluntary and anonymous. Before proceeding, participants were informed about the study purpose, data protection measures and the right to withdraw from the study by contacting the research team with the “timestamp” (date and time) of their participation. Informed consent was obtained by opting-in before continuing to completing the online questionnaire.

### Measurement tools

Participants were asked to complete the SF-12 questionnaire, a widely used and validated instrument for assessing HRQoL [12]. Permission for use was obtained from the copyright owners. The SF-12 assesses two main components of health-related quality of life: the Physical Component Summary (PCS) and Mental Component Summary (MCS) scores. Each component is derived from four sub-scales, covering different aspects of physical and mental well-being. Scores for both PCS and MCS are standardized with a mean of 50 and a standard deviation of 10 for the general population, with scores below 40 considered clinically significant impairment of HRQoL.

PCS reflects physical health and functional limitations, and incorporates the following four sub-scales: physical functioning (PF) which assesses the individual’s ability to perform daily activities, the physical role category (PR), an assessment of the impact of physical conditions on work and daily responsibilities, bodily pain (BP) measuring the intensity of pain and its interference with daily life and finally the sub-scale of general health (GH), reflecting personal perceptions of overall health.

MCS captures emotional wellbeing and mental health status. The scale includes the following four sub-scales: vitality (VT) which assesses energy levels and fatigue, social functioning (SF) which looks at the degree of participation in social activities, the role emotional (RE) sub-scale, examining the extent to which emotional problems interfere with daily activities and responsibilities and, lastly, the mental health sub-category (MH) assessing general mood and psychological distress.

In addition, participants provided demographic and clinical data. In addition to diagnosis and years since diagnosis (YSD), these included gender, age, marital status, occupational status, area of residence, financial difficulties, educational attainment, body mass index (BMI), alcohol consumption, smoking status and physical activity.

BMI was calculated based on participants’ self-reported height and weight and used as a continuous variable in multivariable models. Smoking status was assessed through self-report and categorized as “current smoker”, “past smoker”, and “non-smoker.” Alcohol consumption was evaluated with the question: *“*In the last month, have you consumed any alcohol?” and initially included the categories: “once a month”, “2–3 times in a month”, “once a week”, “2–3 times a week”, and “almost every day”. Alcohol consumption was retained as a categorical variable in multivariable models. Physical activity was assessed through the question: “In the last month, how often did you physically exercise for 30 minutes or more?” with five response options: “Almost every day,” “A few times a week,” “A few times a month,” “Never,” and “I could not exercise.” For multivariable analysis, this variable was treated as an ordinal arithmetic scale. Financial difficulty was assessed through self-report using a single dichotomous question (yes/no), asking participants whether they currently experience any financial difficulties (e.g. in meeting daily household needs or paying bills).

YSD was determined based on the participant’s response to the question: “How many years have passed since you were first diagnosed with your condition?”. In line with established research conventions and the pathophysiological evolution of these conditions, the YSD variable was categorized into four groups: <2 years, 2–10 years, 10–20 years, and > 20 years, to reflect different stages of disease progression and burden.

Diagnosis was determined by participants’ responses to the question: “Have you ever been diagnosed with any of the following conditions?”, providing a list of major RMDs. An open-text option allowed for additional conditions to be listed. For participants recruited from rheumatology clinics, diagnosis was also verified through medical records, whereas for those recruited via the online survey, clinical confirmation was not feasible.

For analysis purpose, the diagnosis variable was categorized in two ways in this study informed by clinical and pathophysiological criteria, as well as the observed distribution of diagnoses in the study sample. The first categorization is a pragmatic analytical framework, grouping RMDs into four broad categories according to broadly shared mechanisms relevant to chronic disease burden and health-related quality of life: [1] Inflammatory rheumatic diseases (Rheumatoid arthritis - RA, ankylosing spondylitis - AS, psoriatic arthritis - PsA, and gout) [2], Connective tissue diseases (Systemic lupus erythematosus - SLE, Sjögren’s syndrome, systemic scleroderma, Behçet’s disease) [3], Soft tissue diseases (fibromyalgia), and [4] Symptomatic peripheral osteoarthritis (knee, hip, and hand osteoarthritis). In particular, the classification distinguishes conditions primarily characterised by persistent inflammatory activity, systemic autoimmune involvement, central sensitisation or degenerative processes.

A second categorization was also applied to assess the impact of select rheumatic diseases independently from the broader clinical grouping they were classified in, while also ensuring adequate sample sizes and statistical stability for multivariable analyses. Specifically, based on their frequency in the sample and research interest, RA and SLE were studied separately leading to the creation of new categories namely, RA, SLE, Fibromyalgia, Other inflammatory rheumatic diseases, Other connective tissue diseases, Symptomatic peripheral osteoarthritis. This approach allowed for a more detailed analysis of the HRQoL impact of the individual conditions.

### Statistical analysis

Descriptive statistics were used to summarize the demographic and clinical characteristics of the sample, whereby continuous variables were presented as means and standard deviations (SD) and categorical variables as frequencies and percentages. SF-12 PCS and MCS scores were calculated according to standard scoring algorithms. Missing responses at the item level were treated as missing and were not imputed. Cases with insufficient item data required for score calculation were excluded from the respective SF-12 score computation. To assess differences in mean PCS and MCS according to RMD categories and YSD, a series of univariable and multivariable linear regression analyses were performed. Multivariable models assessing the association of RMDs categories with HRQoL, adjusted for differences in terms of demographic and clinical characteristics across disease categories: age, gender, marital status, occupational status, area of residence, financial difficulties, educational attainment, body mass index, alcohol consumption, smoking status, physical activity, and YSD. In multivariable models assessing the effect of YSD on HRQoL, the same covariates were included as well as disease categories. Covariates were selected a priori based on clinical relevance and evidence from the literature. Descriptive and exploratory analyses were subsequently conducted to examine their distribution and univariable associations across disease groups, in order to inform appropriate modelling strategies. Depending on their distribution and observed relationships, variables demonstrating approximately linear associations were modelled as continuous terms, whereas non-linear or categorical variables were included using indicator variables. All selected covariates were entered simultaneously into the multivariable models to adjust for potential confounding. Results were reported as beta coefficients (β), capturing differences in mean scores, with 95% confidence intervals (CIs) and p-values. Statistical significance was set at *p* < 0.05.

### Ethics

This study was approved by the Cyprus National Bioethics Committee (Ref: ΕΕΒΚ/ΕΠ/2019/41). Participants provided written informed consent during the on-site data collection. In the online survey, information about the study including the type of data collected, the purpose of the study, and the intended use of the data was provided on the landing page. Respondents had to opt-in by selecting “yes” to the informed consent statements before proceeding to the survey. Participation in both on-site and online surveys was voluntary and anonymous, safeguarding the right to discontinue or withdraw at any time. For on-site visits to the outpatient departments and Rheumatology Clinics at General Hospitals, additional approval was obtained from the Cyprus State Health Services Organization, as per appropriate procedure.

## Results

### Socio-demographic characteristics

Socio-demographic and clinical characteristics of the study sample are presented in Tables 1 and 2. A total of 789 individuals participated in the study, with 60% recruited through onsite visits, while the remaining 40% via completing the online questionnaire. The majority of participants were women (76.6%), and most were married or cohabiting with a partner (71.8%). The average age of participants was 55.4 years (SD = 13.2), with the largest percentage in the age category 40–49 years of age (29.6%), followed by participants under 39 years of age (19.4%).

RMDs were initially grouped into 4 categories with the largest proportion in the inflammatory rheumatic diseases category (50.9%), followed by connective tissue disorders (23.8%), soft tissue diseases (14. 1%) and symptomatic peripheral arthritis (11.2%). For the purposes of analysis, a secondary classification was also applied to allow more detailed focus on specific diseases with higher frequency in the sample: RA (30.4%), other inflammatory diseases (20.5%), SLE (14.7%), other connective tissue diseases (9.1%), Fibromyalgia (14.1%) and Symptomatic peripheral osteoarthritis (11.2%). Participants in the study had a wide range of disease durations, reflecting varying stages of living with an RMD. The largest proportion (39.0%) had been diagnosed between 2 and 10 years prior to the study. This was followed by those diagnosed 10–20 years ago (26.8%), more than 20 years ago (17.2%), and within the past 2 years (17.0%).

**Table 1:** Socio-demographic characteristics of study participants

Variable	Category	*n* (%)
**Gender**	Female	604 (76.6%)
	Male	184 (23.4%)
Age	18–29	38 (4.9%)
	30–39	115 (14.8%)
	40–49	230 (29.6%)
	50–59	190 (24.1%)
	60–69	134 (17.2%)
	> 70	71 (9.1%)
Marital Status	Married or living with partner	564 (71.8%)
	Not Married	85 (10.8%)
	Divorced	95 (12.1%)
	Widower	42 (5.3%)
Education	None or Primary	47 (6.0%)
	Secondary	247 (31.5%)
	Tertiary-College	201 (25.6%)
	Tertiary-University	289 (36.9%)
District of usual residence	Nicosia	369 (46.8%)
	Limassol	194 (24.6%)
	Larnaca	159 (20.2%)
	Paphos	39 (4.9%)
	Ammochostos	28 (3.5%)
Occupational	Full-time employed	417 (53.1%)
	Part-time employed	60 (7.6%)
	Unemployed	35 (4.5%)
	Retired	167 (21.2%)
	Student/unpaid worker	9 (1.1%)
	Homemaker, Parent, Caregiver	31 (3.9%)
	Permanent or temporary disability due to illness	67 (8.5%)
Any Financial difficulties	Yes	258 (33.0%)
	No	525 (67.0%)

**Table 2:** Clinical and other health-related characteristics of study participants

Variable	Category	*n* (%)
Alcohol Intake	Never	324 (41.4%)
	Once in a month	205 (26.2%)
	2–3 times in a month	141 (17.9%)
	Once a week	65 (8.3%)
	2–3 times a week or more	48 (6.1%)
Physical activity	Almost every day	81 (10.3%)
	A few times a week	242 (30.8%)
	A few times a month	155 (19.7%)
	Never	194 (24.7%)
	I could not exercise	113 (14.4%)
Smoking	Smoker	162 (20.5%)
	Past Smoker	93 (11.8%)
	Non-smoker	533 (67.6%)
Body Mass Index	Underweight	41 (5.2%)
	Healthy Weight	321 (41.3%)
	Overweight	268 (34.5%)
	Obesity	147 (18.6%)
Diseases categories (First categorization)	Inflammatory Rheumatic Diseases	402 (50.9%)
	Connective Tissue Diseases	188 (23.8%)
	Symptomatic peripheral osteoarthritis	88 (11.2%)
	Soft Tissue Diseases (Fibromyalgia)	111 (14.1%)
Diseases categories (Second categorization)	Other Inflammatory Rheumatic Diseases	162 (20.5%)
	Rheumatoid Arthritis	240 (30.4%)
	Other Connective Tissue Diseases	72 (9.1%)
	Systemic Erythematosus Lupus	116 (14.7%)
	Symptomatic peripheral osteoarthritis	88 (11.2%)
	Fibromyalgia	111 (14.1%)
Years since diagnosis (YSD)	Less than 2 years	131 (17.0%)
	2 to less than 10 years	301 (39.0%)
	10 to less than 20 years	207 (26.8%)
	20 years or more	133 (17.2%)

### Overall, HRQoL

Table 3 presents descriptive statistics for the continuous PCS and MCS scores, as well as the distribution of participants across different HRQoL categories. High variability was observed in both component scores, reflecting the diverse impact of RMDs on physical and mental health. The majority of participants (57.4%) were classified in the low PCS category (< 40), with an additional 17.6% in the medium-low [13–23], while only 16.2% reported high PCS scores (> 60), indicating a substantial proportion experiencing physical health limitations. MCS scores appeared relatively better compared to PCS scores, even though a high proportion of participants still scored low. Specifically, 38.7% of participants were categorized as low MCS (< 40), while an additional 24.8% fell into the medium-low range.

**Table 3:** Summary of HRQoL scores (Overall, PCS and MCS)

**Measure**	**Overall HRQoL**	**PCS**	**MCS**
Mean (SD)	41.99 (18.97)	37.63 (20.28)	46.34 (20.41)
Median (IQR)	40.63 (26.56)	37.50 (29.68)	43.75 (28.13)
**HRQoL categorization**			
**HRQoL category**		**PCS (N**,** %)**	**MCS (N**,** %)**
Low *(< 40)*		453(57.4%)	305 (38.7%)
Medium-Low *(40 to less than 50)*		139 (17.6%)	196 (24.8%)
Medium-High *(50 to less than 60)*		128 (16.2%)	102 (12.9%)
High *(> 60)*		69 (8.8%)	186 (23.6%)

Descriptive comparisons between clinic-recruited and online-recruited participants showed no statistically significant differences in physical or mental health summary scores, supporting the pooled analysis of the overall sample (Supplementary Table S1).

**Fig. 1:**
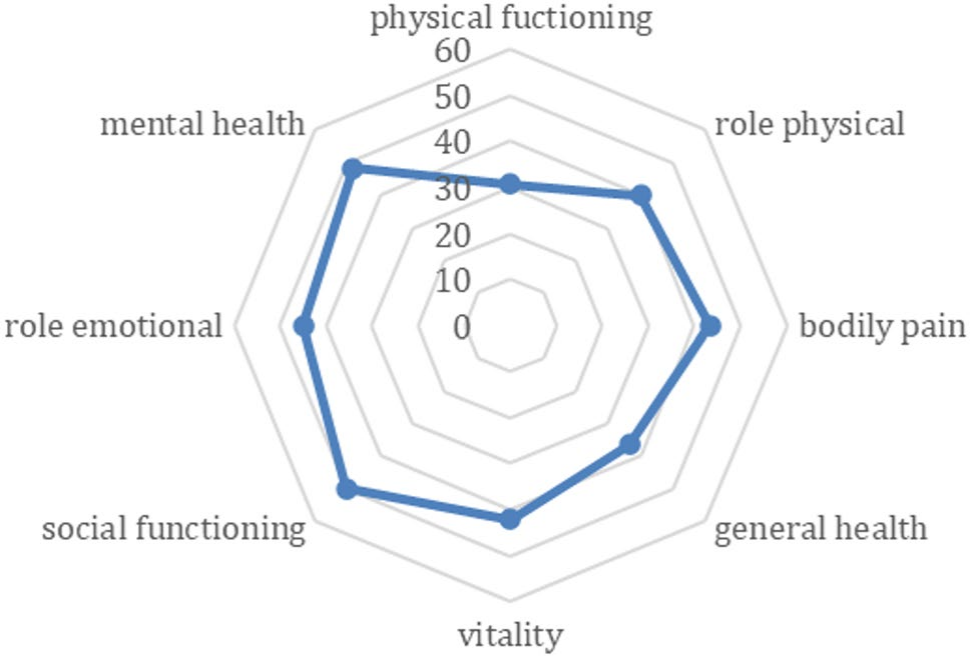
Mean scores and SD for SF-12 subscales among participants. This figure presents the mean and SD of scores across the eight subscales of the SF-12 questionnaire

The radar plot in Fig. 1 shows the mean values for all subscales of the SF-12. The sub-scales Social Functioning (SF) and Mental Health (MH) recorded the highest scores on average, with mean values of 50.19 (SD: 27.56) and 48.19 (SD: 21.09), respectively, In contrast, sub-scales Physical Functioning (PF) with mean 30.67 (SD:21.89) and General Health (GH) with mean 36.47 (SD:22.24) show the lowest scores.

### Differences in HRQoL across broad disease category groups

**Fig. 2:**
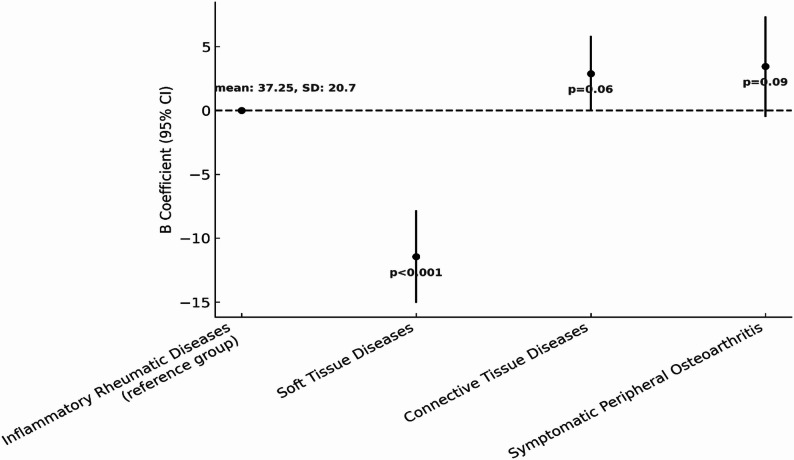
Multivariable regression model examining the association between RMDs and PCS. The figure presents comparison of PCS scores across RMDs: inflammatory RMDs (reference), connective tissue diseases, soft tissue diseases (fibromyalgia), and symptomatic peripheral osteoarthritis. Adjusted for demographic and lifestyle variables

**Fig. 3:**
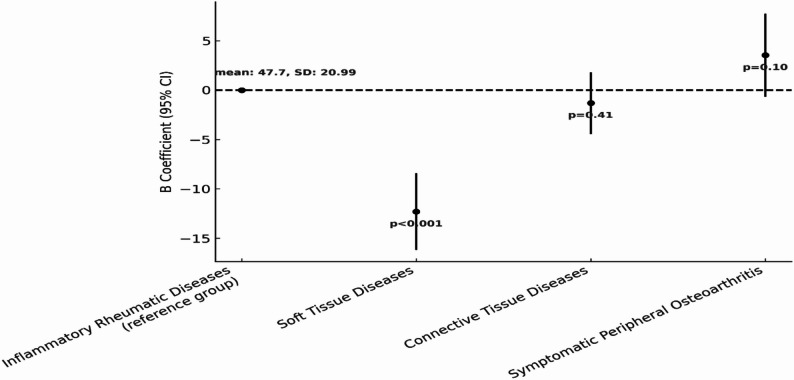
Multivariable regression model examining the association between RMDs and MCS. The figure presents comparison of MCS scores across RMDs: inflammatory RMDs (reference), connective tissue diseases, soft tissue diseases (fibromyalgia), and symptomatic peripheral osteoarthritis. Adjusted for demographic and lifestyle variables

To explore differences in HRQoL across RMDs categories, multivariable linear regression analyses were conducted for both PCS and MCS scores, adjusting for demographic and clinical characteristics. Figures 2 and 3 illustrate the estimated differences in PCS and MCS, respectively, using the inflammatory rheumatic diseases group as the reference. Based on results of the regression analysis, participants with soft tissue diseases had the lowest scores on average in both PCS (B = -11.45, 95% CI: -15.06, -7.83; *p* < 0.001) and MCS (B = -12.31, 95% CI: -16.18, -8.4, *p* < 0.001). Compared to inflammatory RMDs (reference group), connective tissue diseases category showed a slightly improved PCS on average, but marginally short of statistical significance (B = 2.89, 95% CI: -0.06, 5.84, *p* = 0.06). In contrast, for MCS, there was a slight non-significant lower score (B = -1.30, 95% CI: -4.47, 1.8, *p* = 0.41). Lastly, participants in the symptomatic peripheral osteoarthritis category recorded higher HRQoL scores for both PCS (B = 3.45, 95% CI: -0.50, 7.36, *p* = 0.09) and MCS (B = 3.53, 95% CI: -0.66, 7.73, *p* = 0.1) compared to inflammatory RMDs (reference group), but observed differences were not statistically significant.

### Differences in HRQoL across specific disease categories

**Fig. 4:**
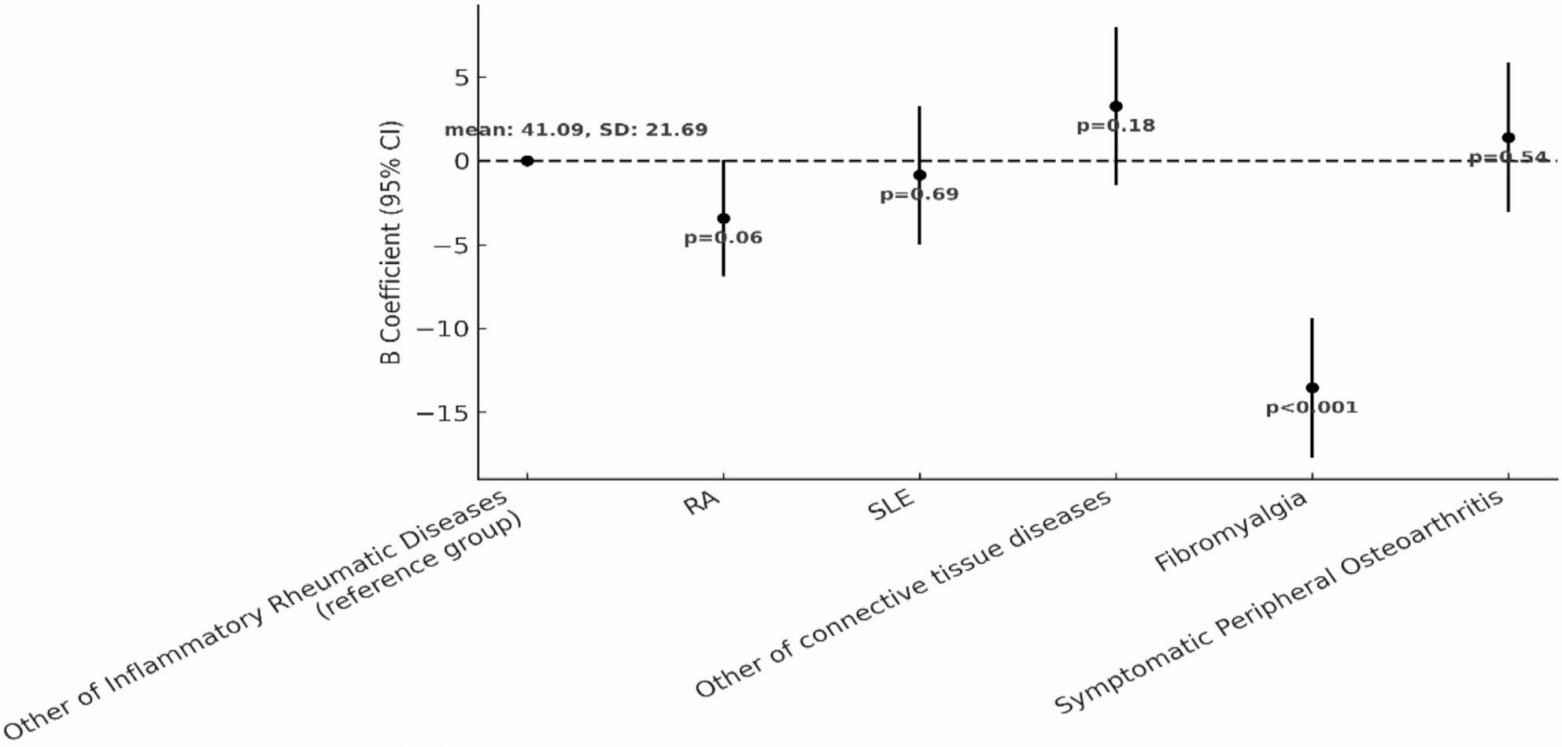
Multivariable regression model examining the association between Major Rheumatic Disease Subtypes and PCS. The figure presents comparison of PCS scores across specific diagnoses (RA, SLE, Fibromyalgia, other inflammatory rheumatic diseases (reference), other connective tissue diseases, symptomatic peripheral osteoarthritis. Adjusted for demographic and lifestyle variables

**Fig. 5:**
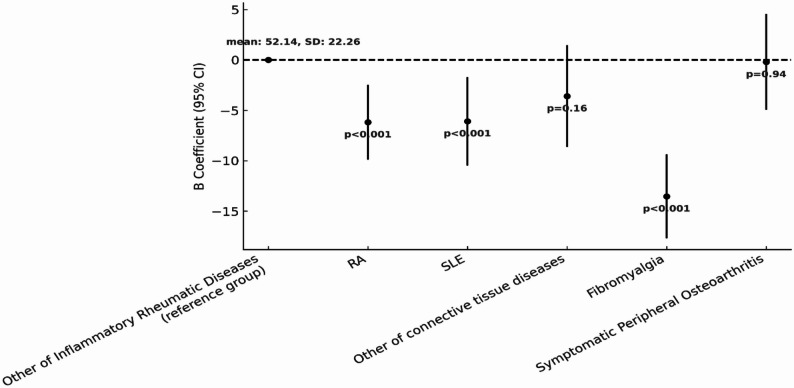
Multivariable regression model examining the association between Major Rheumatic Disease Subtypes and MCS. The figure presents comparison of MCS scores across specific diagnoses (RA, SLE, Fibromyalgia, other inflammatory rheumatic diseases (reference), other connective tissue diseases, symptomatic peripheral osteoarthritis. Adjusted for demographic and lifestyle variables

To further explore differences in HRQoL across individual conditions, a second categorization of RMDs was used. A multivariable regression analysis was conducted, setting the category of “other inflammatory RMDs” (which include ankylosing spondylitis, psoriatic arthritis, and gout) as the reference group. Figures 4 and 5 present the results for PCS and MCS respectively.

Individuals with fibromyalgia are those who report the lowest HRQoL both with regard to PCS (B = -13.54, 95% CI: -17.71, -9.37, *p* < 0.001) and MCS (B = -16.02, 95% CI: -20.47, -11.58, *p* < 0.001). RA also shows statistically significantly lower MCS (B = -6.17, 95% CI: -9.89, -2.46, *p* < 0.001-) compared to the reference group. While the PCS scores were also lower on average, the observed difference did not reach statistical significance at the 5% level (B = -3.42, 95% CI: -6.90, 0.07, *p* = 0.06). Similarly, participants with SLE showed statistically significantly poorer MCS scores compared to the reference group (B = -6.08, 95% CI: -10.47, -1.70, *p* < 0.001), while for PCS no significant difference was observed compared to the reference group (B = -0. 84, 95% CI: -4.97, 3.30, *p* = 0.69).

For those in the symptomatic peripheral osteoarthritis diseases category, there were no significant differences in either PCS (B = 1.39, 95% CI: -3.06, 5.84, *p* = 0.54) or MCS (B = -0.19, 95% CI: -4.94, 4.55, *p* = 0.94). Finally, the category of other connective tissue diseases showed better results in PCS (B = 3.26, 95% CI: -1.48, 8.00, *p* = 0.18) but results for MCS were in the opposite direction indicating worse mental health outcomes compared to the reference group (B = -3.60, 95% CI: -8.65, 1.46, *p* = 0.16). However, these differences were not statistically significant.

### Differences in HRQoL by YSD

To examine the association between disease duration and HRQoL, a multivariable regression analysis was conducted with YSD as the main independent variable, with reference group set as individual diagnosed between 2 to less than 10 years ago.

**Fig. 6:**
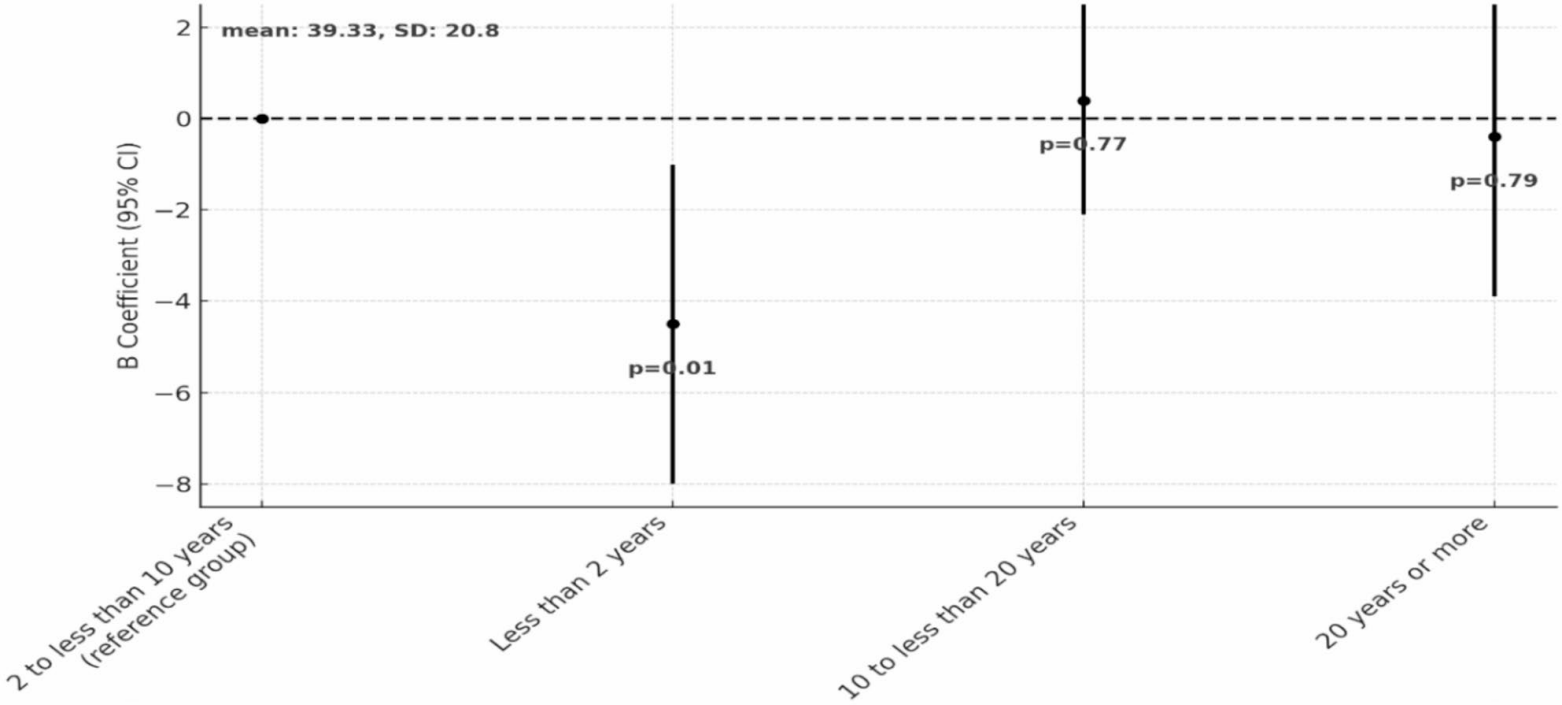
Multivariable regression model examining the association between YSD and PCS. The figure presents comparison of PCS scores across disease duration categories: Less than 2 years, 2 to less than 10 years (reference), 10 to less than 20 years, 20 years or more

**Fig. 7:**
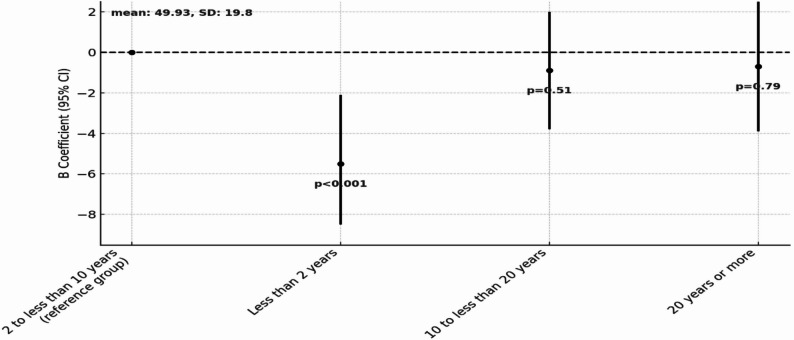
Multivariable regression model examining the association between YSD and MCS. The figure presents comparison of MCS scores across disease duration categories: Less than 2 years, 2 to less than 10 years (reference), 10 to less than 20 years, 20 years or more

Participants with the shortest diseased duration (< 2 years since diagnosis) appear to face the greatest challenges in both physical and mental health with significantly lower values in both PCS (B = -4.53, 95% CI: -8.05, -1.00, *p* = 0.01) and MCS (B = -5.51, 95% CI: -9.28, -1.75, *p* < 0.001) compared to the reference group. In contrast, for participants with YSD between 10 and less than 20 years, there was no statistically significant difference from the reference group in either PCS (B = 0.44, 95% CI: -2.49, 3.36, *p* = 0.77) or MCS (B = -1.05, 95% CI: -4.19, 2.09, *p* = 0.51). A similar pattern was observed for the group with the longest disease duration (20 years of more), where mean scores were comparable to the reference group for both PCS (B = -0.48, 95% CI: -3.99, 3.04, *p* = 0.79) and MCS (B = -0.90, 95% CI: -4.61, 2.81, *p* = 0.63). The distribution of PCS and MCS scores across different disease duration groups is presented in Figs. 6 and 7, respectively.

## Discussion

This study highlights the significant impact of RMDs on HRQoL, despite the considerable variability observed among broad or specific disease categories. Overall, PCS was more severely affected than MCS although both domains showed considerable impairment. Inflammatory RMDs, which comprise the largest proportion of the study sample, were associated with moderate levels of HRQoL impairment with physical health being the most affected domain. Connective tissue diseases showed a similar overall burden, though mental health was more significantly impacted. In the more detailed classification, fibromyalgia emerged as the most challenging with worse outcomes in both physical and mental health. RA and SLE were also associated with worse mental health outcomes compared to other inflammatory diseases. Conversely, peripheral osteoarthritis recorded the highest by comparison HRQoL in both domains.

Regarding disease duration, newly diagnosed individuals experienced significantly poorer HRQoL in both physical and mental components of HRQoL components. Longer-standing disease (≥ 10 years) did not differ significantly, suggesting a possible adaptation or plateau over time. These findings emphasize the importance of condition-specific interventions particularly at the early stages and tailored support mechanisms that consider the variability and complexity of RMDs.

### Comparisons of HRQoL in RMDs with control groups or general population

RMDs represent a major global health burden and are consistently associated with substantial impairment in HRQoL, affecting both physical and mental health compared with the general population [24]. A number of studies in the literature compared individuals with RMDs to control groups or general population samples. These studies consistently report lower HRQoL across multiple domains [25–29]. Disease-specific investigations focusing on conditions such as rheumatoid arthritis, ankylosing spondylitis and SLE similarly demonstrate significant HRQoL deterioration [30–33]. In the absence of a general population control group in the present study, comparisons with population or control samples are discussed solely to provide contextual background from the literature and should not be interpreted as direct findings derived from this analysis.

Evidence from studies using the SF-12 questionnaire highlights markedly reduced physical health scores among people living with RMDs [25, 30]. Chronic pain, stiffness, inflammation and functional limitations, common features in conditions such as RA, ankylosing spondylitis and psoriatic arthritis are key contributors to physical disability and reduced mobility, underscoring the need for targeted interventions to support functional health [5, 26, 34].

Although mental health outcomes are often less severely affected than physical health, anxiety, depression and fatigue remain prevalent among individuals with RMDs [35]. The cumulative burden of chronic pain and disability over the disease course further highlights the interrelated nature of physical and mental health domains [36, 37].

Within the broader literature, a number of research studies have also explored differences in HRQoL across RMD categories, illustrating heterogeneity within the broader spectrum of RMDs [2, 4, 6]. In line with this literature, the present study focuses on internal comparisons across RMDs, aiming to elucidate disease-specific patterns and variation in HRQoL, rather than to quantify differences relative to the general population.

### Comparison of HRQoL between RMDs categories

This study highlights the difference in HRQoL between categories of RMDs. People with fibromyalgia, due to the chronic pain and fatigue resulting from central sensitization, are the most severely affected. HRQoL levels for people with fibromyalgia have significantly lower values in both physical and mental health, not only when compared with the general population [38] and control groups [39, 40] but also in comparison with other RMDs [6, 41–44].

Inflammatory diseases, such as RA, due to the systemic inflammation, have a significant impact on physical functioning and mental health [30, 31, 45–47]. Connective tissue diseases, which involve multi-organ damage, show more variability in their impact on HRQoL, possibly depending on variable disease severity [28, 32, 48, 49]. Peripheral osteoarthritis, which involves local joint degeneration, primarily affects physical mobility, with comparatively less systemic effects [34].

The pervasive and debilitating effect of fibromyalgia on HRQoL is supported by the findings of the present survey, and confirmed in previous studies [38, 41]. Central sensitization mechanisms uniquely exacerbate both physical and mental health impairments [13, 50]. The burden of fibromyalgia is both severe and multifaceted, highlighting the critical need for individualized, multidisciplinary interventions to address it. As opposed to inflammatory RMDs, which primarily affect physical functioning due to systemic inflammation and joint damage caused as a result of disease progression [30, 46, 47], the central sensitization mechanisms of fibromyalgia exacerbate chronic pain and fatigue, significantly reducing physical health, while worsening the psychological well-being of individuals [6, 14, 43, 44]. Inflammatory RMDs, although associated with reduced PCS and MCS, show comparatively better outcomes probably due to targeted anti-inflammatory therapies and disease modification interventions [15]. Connective tissue diseases, due to their systemic and multifactorial nature, tend to affect MCS to a greater extent, while also showing a moderate effect on PCS, adding to the psychological burden of systemic complications [16, 17]. Peripheral osteoarthritis, with its localized effects, shows generally better PCS and MCS, compared to fibromyalgia and connective tissue diseases, yet HRQoL remains reduced due to pain and mobility limitations [4, 18].

To gain a better understanding of how RMDs affect HRQoL, this study also employed a secondary categorization strategy, assessing RA and SLE separately from their respective broader subgroups. This approach aimed to capture the distinct ways in which these conditions impact both physical and mental health.

Fibromyalgia remains, even with this separation, the condition with the greatest deterioration in both physical health and mental well-being, further supporting the negative impact of central sensitization mechanisms in pain and psychological well-being [18, 44]. Compared to other inflammatory diseases, RA had the greatest physical impact, highlighting the role of systemic inflammation and joint destruction. Studies have implicated chronic pain and the systemic effects of RA as key factors leading to physical disability [5, 19]. Other studies have noted that the improved HRQoL for ankylosing spondylitis and psoriatic arthritis is attributed to the effectiveness of available biological therapies [20, 21, 51]. For RA, early use of biologic drugs and disease-modifying antirheumatic drugs (DMARDs) is critical to mitigate the effects of its systemic and multifactorial nature [22, 23]. SLE showed the lowest PCS and MCS scores among connective tissue diseases, reflecting its severe systemic and psychological burden [32]. Other connective tissue diseases showed relatively better PCS but poorer MCS compared to inflammatory diseases, underlining their unique psychological challenges [4, 41]. Peripheral osteoarthritis showed similar PCS and MCS outcomes to inflammatory diseases, highlighting its localized yet chronic HRQoL burden [26, 34, 52].

### Targeted approaches to improve HRQoL in RMDs

Implementing targeted interventions that address underlying pathophysiological mechanisms is critical. Due to the heterogeneity of RMDs both in terms of symptom presentation and severity, as well as their systemic involvement, interventions must be tailored to the unique needs of each condition and disease progression. For fibromyalgia, interventions should focus on chronic pain and central sensitization, which are key drivers of both physical and mental health impairments. Inflammatory RMDs are directly affected by systemic inflammation and joint distraction, making early management of inflammation a critical factor for improving prognosis. Prompt initiation of DMARDs and biologic agents helps slow disease progression, reduce joint damage, and improve overall HRQoL [22, 23, 53]. Moreover, the implementation of interdisciplinary and integrated care models can address the multifaceted challenges posed by these conditions. A combination of physiotherapy, patient education, and psychological support has been shown to significantly improve physical health and overall well-being [54]. Connective tissue diseases, due to their systemic and multi-organ complications, require comprehensive care approaches that focus on both physical health and mental resilience. Peripheral osteoarthritis benefits from localized interventions targeting joint-specific pain and mobility; however, its impact on HRQoL necessitates a broader holistic management approach. Recent evidence support that while targeted interventions, such as physiotherapy and intra-articular injections, can effectively relieve joint-specific symptoms, broader approaches that include lifestyle modifications and patient education are essential to improve overall HRQoL [51, 55].

### Disease duration and HRQoL

The effect of disease duration on the HRQoL of people with RMDs has been well studied, providing valuable insights into how physical and mental health evolve over time. The present study aligns with previous findings that suggest that early stages are characterized by significant physical and psychological challenges. The acute impact of inflammation, disease activity and the burden of adjustment to chronic disease during the initial period have been linked to declines in HRQoL [56, 57]. Poor HRQoL in the early stages is also closely linked to delays in diagnosis and initiation of appropriate treatment [58, 59], which can intensify both physical suffering and psychological distress, a common issue in many RMDs, particularly fibromyalgia, inflammatory and connective tissue diseases.

In the mid-stage of the disease, improved levels of HRQoL are often observed. Recent literature emphasizes the role of effective disease adaptation and management strategies in enhancing patient outcomes [54]. Notably, the early implementation of targeted interventions to patients with inflammatory RMDs has been shown to reduce long-term disability and significantly improve both physical and mental health [51]. In the later stages of the disease, particularly among individuals who have lived with the condition for over two decades, functional stability is frequently reported. This stability may reflect the use of tailored therapeutic approaches, aimed at managing disease activity, as well as the cumulative effect of coping mechanisms [60, 61]. However, despite these adaptive strategies, persistently low PCS and MCS scores relative to population norms underscore the continued burden of comorbidities and challenges in maintaining physical and mental well-being [41].

From a public health perspective, these findings highlight the importance of timely identification and coordinated care for individuals living with RMDs, particularly during the early stages of disease. The substantial variability observed in HRQoL across diagnostic categories and stages underscores the value of person-centred and multidisciplinary approaches to chronic disease management. In healthcare systems undergoing organizational transition, such as Cyprus, population-based HRQoL data may provide useful contextual information to inform future discussions around service planning and patient support. Incorporating HRQoL indicators into routine assessment has the potential to complement clinical evaluation and to enhance understanding of patient needs, although the present study was not designed to evaluate healthcare system performance or policy impact.

### Study limitations

The study has some limitations that should be acknowledged. First, the absence of clinical confirmation of diagnosis for participants recruited through online channels represents an important limitation, as reliance on self-reported information may have led to diagnostic misclassification. This limitation also extends to other self-reported clinical variables, such as YSD. It should be noted though that as many as 90% of the online participants stated that diagnosis was given by a Rheumatologist. Second, although a number of participants reported multiple rheumatic diagnoses (approximately 12%), separate overlap subgroups were not considered due to the small numbers involved, which would have resulted in imprecise estimates. Consequently, diagnostic overlap, particularly among online respondents, may have influenced HRQoL comparisons. Nevertheless, descriptive comparisons between clinic-recruited and online-recruited participants revealed no statistically significant differences in physical or mental health summary scores, suggesting a broadly comparable HRQoL profile across recruitment groups.

Third, cross-sectional study design captures HRQoL at a single point in time and therefore does not allow the assessment of within-person changes or temporal trajectories of the effect of RMDs on HRQoL, which could be evaluated with longitudinal studies. In addition, comparisons across disease-duration strata may be influenced by cohort effects and survivor bias, as individuals with longer disease duration may represent patients with better adaptation or less severe disease. Furthermore, despite adjusting for key covariates, important variables such as type of treatment, adherence to therapy and disease activity have not been considered. Consequently, findings should be interpreted as descriptive associations rather than evidence of longitudinal change or causal effects. Future studies should aim to incorporate these elements using longitudinal or repeated cross-sectional designs, ideally incorporating detailed clinical and treatment-related measures, to provide a more comprehensive understanding of HRQoL trajectories over the course of RMDs disease trajectories. Norm-based analysis was not applied to the SF-12 results due to lack of established normative data for the Cypriot population. This limits direct comparability with other population-based studies that use national standard norms. Thus, only the SF-12 component summary scores were used in this study to enable meaningful internal comparisons between RMDs categories and YSD. Although the SF-12 allows broad comparability across conditions, future research could benefit from incorporating disease-specific HRQoL instruments to capture dimensions such as pain, fatigue, and functional impairment in greater detail.

Finally, the mixed recruitment approach represents both a limitation and a strength of this study. Recruitment through rheumatology clinics may tend to overrepresent individuals with more active disease or ongoing engagement with healthcare services. The parallel online recruitment strategy facilitated the inclusion of individuals with different patterns of disease management, including those not currently in regular clinical follow-up or managed in less intensive care settings. This consideration is particularly relevant in the Cypriot context, where the recent transition to the General Healthcare System (GeSY) has been accompanied by variable readiness of specialist services operating under high demand due to existing caseloads and new referrals from family physicians. Together, these approaches allowed for the capture of a broader spectrum of people living with RMDs.

## Conclusion

This study confirms the significant impact of RMDs on HRQoL, highlighting substantial variability depending on diagnosis and disease duration. Fibromyalgia presents the greatest challenges both physically and psychologically. A considerable burden is observed in both inflammatory diseases and connective tissue diseases. Examining HRQoL outcomes across RMDs within a broad pathophysiological framework, reflecting dominant mechanisms such as inflammatory activity, systemic immune involvement, degenerative processes, or central sensitization may help contextualize observed differences between disease categories and inform future efforts to develop more targeted, patient-centred interventions. Importantly the findings also highlight the substantial HRQoL burden observed during the early phase following diagnosis, underscoring the importance of timely initiation of treatment and adequate support at the onset of the disease. In healthcare systems undergoing organizational transition, the pronounced HRQoL deficits observed in this study point to substantial unmet needs and ongoing challenges faced by people living with RMDs. While the present findings do not permit evaluation of health system performance, they underscore the importance of timely identification, coordinated and patient-centred care, and sustained support across the disease course. In this context, HRQoL data may provide valuable contextual signals to inform future discussions on care pathways and the integration of patient-reported outcomes into routine practice.

## Supplementary Information

Below is the link to the electronic supplementary material.

Supplementary Material 1

## Data Availability

The datasets used and/or analyzed during the current study are available from the corresponding author on reasonable request.

## Declarations

All participants provided informed consent in line with the recruitment method. For clinic-based data collection, participants received written information on the standard form of the Cyprus National Bioethics Committee and provided signed consent prior to participation. In the online survey, participants were presented with detailed information using an adapted format of the same consent form and provided informed consent electronically by agreeing to the consent declaration before survey initiation. The study was conducted in accordance with the ethical standards laid down in the 1964 Declaration of Helsinki and its later amendments.

Clinical trial number: not applicable.

## Abbreviations

BMI: Body mass index
DMARDs: Disease-modifying antirheumatic drugs
GH: General health
HRQoL: Health-related quality of life
MCS: Mental Component Score
MH: Mental health
PCS: Physical Component Score
PF: Physical functioning
RA: Rheumatoid arthritis
RE: Role emotional
RMD(s): Rheumatic and musculoskeletal disease(s)
SF: Social functioning
SF-12: Short Form 12 questionnaire
SLE: Systemic lupus erythematosus
VT: Vitality
WHO: World Health Organization
YSD: Years since diagnosis

## References

[1] Cross M, Smith E, Hoy D, Carmona L, Wolfe F, Vos T, et al. The global burden of rheumatoid arthritis: estimates from the global burden of disease 2010 study. Ann Rheum Dis. 2014;73(7):1316–22. 24550173 10.1136/annrheumdis-2013-204627

[2] Michaud K, Solomon DH, Zhang Y, Maher N, Mikuls TR. Depression, anxiety and cognitive function in persons with inflammatory rheumatic disease compared to osteoarthritis: a population-based study. RMD Open. 2023;10(4):e004808.

[3] Matcham F, Scott IC, Rayner L, Hotopf M, Kingsley GH, Norton S, et al. The impact of rheumatoid arthritis on quality-of-life assessed using the SF-36: A systematic review and meta-analysis. Semin Arthritis Rheum. 2014;44(2):123–30. 24973898 10.1016/j.semarthrit.2014.05.001

[4] Salaffi F, Di Carlo M, Carotti M, Farah S, Ciapetti A, Gutierrez M. The impact of different rheumatic diseases on health-related quality of life: A comparison with a selected sample of healthy individuals using SF-36, EQ-5D, and SF-6D utility values. Acta Biomed. 2018;89(1):131–9. 10.23750/abm.v89i4.7298 PMC6502108 30657123

[5] Salaffi F, Carotti M, Gasparini S, Intorcia M, Grassi W. Health-related quality of life in rheumatoid arthritis, ankylosing spondylitis, and psoriatic arthritis: A comparison with a selected sample of healthy people. Health Qual Life Outcomes. 2009;7:25. 19296831 10.1186/1477-7525-7-25 PMC2674445

[6] Ovayolu N, Ovayolu Ö, Karadag G. Health-related quality of life in ankylosing spondylitis, fibromyalgia syndrome, and rheumatoid arthritis: A comparison with a selected sample of healthy individuals. Clin Rheumatol. 2011;30(5):655–64. 21057839 10.1007/s10067-010-1604-2

[7] Müller R, Kallikorm R. An integrated approach to the treatment of rheumatic diseases: the role of psychological factors. Rheumatol Int. 2024;44(5):789–98. 10.1007/s00296-024-05728-9 39400563

[8] Covic T, Cumming SR, Pallant JF, Manolios N, Emery P, Conaghan PG, et al. Depression and anxiety in patients with rheumatoid arthritis: prevalence rates based on a comparison of the depression, anxiety and stress scale (DASS) and the hospital anxiety and depression scale (HADS). BMC Psychiatry. 2012;12:6. 22269280 10.1186/1471-244X-12-6 PMC3285517

[9] Kojima M, Kojima T, Ishiguro N, Oguchi T, Oba M, Tsuchiya H, et al. Predictors of impaired quality of life in patients with rheumatic diseases. Clin Rheumatol. 2016;35(6):1521–30. 26700441 10.1007/s10067-015-3155-z

[10] Chiu YM, Lai MS, Lin HY, Lang HC, Lee LJ, Wang JD. Disease activity affects all domains of quality of life in patients with rheumatoid arthritis and is modified by disease duration. Clin Exp Rheumatol. 2014;32(6):898–903. 25189095

[11] Yip K, Navarro-Millán I. Racial, ethnic, and healthcare disparities in rheumatoid arthritis. Curr Opin Rheumatol. 2021;33(2):117–21. 33394602 10.1097/BOR.0000000000000782 PMC8009304

[12] Ware J Jr, Kosinski M, Keller SD. A 12-item short-form health survey: construction of scales and preliminary tests of reliability and validity. Med Care. 1996;34(3):220 – 33. 10.1097/00005650-199603000-00003. PMID: 8628042. 10.1097/00005650-199603000-00003 8628042

[13] Nijs J, Torres-Cueco R, van Wilgen CP, Girbes EL, Struyf F, Roussel N, et al. Central sensitisation in chronic pain conditions: implications for clinical practice. Lancet Rheumatol. 2021;3(6):e383–92. 38279393 10.1016/S2665-9913(21)00032-1

[14] Zhang Y, Liang D, Jiang R, Ji X, Wang Y, Zhu J, et al. Clinical, psychological features, and quality of life of fibromyalgia patients: A cross-sectional study of Chinese sample. Clin Rheumatol. 2017;37(4):527–37. 29027043 10.1007/s10067-017-3872-6

[15] Min HK, Kim SH, Kim HR, Lee SH. Therapeutic utility and adverse effects of biologic disease-modifying anti-rheumatic drugs in inflammatory arthritis. Int J Mol Sci. 2022;23(22):13913. 36430392 10.3390/ijms232213913 PMC9692587

[16] Segal B, Bowman SJ, Fox PC, Vivino FB, Murukutla N, Brodscholl J, et al. Primary Sjögren’s syndrome: health experiences and predictors of health quality among patients in the united States. Health Qual Life Outcomes. 2009;7:46. 19473510 10.1186/1477-7525-7-46 PMC2693523

[17] Chaigne B, Finckh A, Alpizar-Rodriguez D, Courvoisier D, Ribi C, Chizzolini C. Differential impact of systemic lupus erythematosus and rheumatoid arthritis on health-related quality of life. Qual Life Res. 2017;26(7):1767–75. 28285445 10.1007/s11136-017-1534-4

[18] Leme MOP, Yuan SLK, Magalhães MO, Meneses SRF, Marques AP. Pain and quality of life in knee osteoarthritis, chronic low back pain and fibromyalgia: A comparative cross-sectional study. Reumatismo. 2019;71(2):68–74. 31309776 10.4081/reumatismo.2019.1104

[19] Ristic B, Carletto A, Fracassi E, Pacenza G, Zanetti G, Pistillo F, et al. Comparison and potential determinants of health-related quality of life among rheumatoid arthritis, psoriatic arthritis, and spondyloarthritis: A cross-sectional study. J Psychosom Res. 2023;175:111512. 37844390 10.1016/j.jpsychores.2023.111512

[20] Maruotti N, Cantatore FP. Impact of biological therapy on spondyloarthritis. Eur J Clin Pharmacol. 2014;70:1021–7. 24909930 10.1007/s00228-014-1706-x

[21] Coates LC, Helliwell PS. Biological therapy for psoriatic arthritis: current state and future perspectives. Rheumatol Int. 2024;44(3):253–61. 10.1007/s00296-024-05722-1 39311915

[22] Smolen JS, Aletaha D, Bijlsma JW, Breedveld FC, Boumpas D, Burmester G, et al. Treating rheumatoid arthritis to target: recommendations of an international task force. Ann Rheum Dis. 2010;69(4):631–7. 20215140 10.1136/ard.2009.123919 PMC3015099

[23] Singh JA, Saag KG, Bridges SL, Akl EA, Bannuru RR, Sullivan MC, et al. 2015 American college of rheumatology guideline for the treatment of rheumatoid arthritis. Arthritis Care Res (Hoboken). 2016;68(1):1–25. 26545825 10.1002/acr.22783

[24] Rasu RS, Khan NS, Holmes HM, Marupuru S, Rivera-Colon G, Syed MH. The association between social determinants of health and all-cause mortality in rheumatoid arthritis: a population-based cohort study. BMC Public Health. 2025;25(1):706.39979885

[25] Salaffi F, Di Carlo M, Farah S, Carotti M, Gutierrez M. The impact of different rheumatic diseases on health-related quality of life: A comparison with a general population of Italy. Health Qual Life Outcomes. 2019;17(1):82. 30657123 10.23750/abm.v89i4.7298 PMC6502108

[26] Ferreira AH, Godoy PB, Oliveira NR, Diniz R, Diniz RE. Investigation of depression, anxiety and quality of life in patients with knee osteoarthritis. Rev Bras Reumatol. 2015;55(5):434–8. 26198010 10.1016/j.rbr.2015.03.001

[27] Abu Al-Fadl EM, Ismail MA, Thabit M, El-Serogy Y. Assessment of health-related quality of life, anxiety, and depression in patients with early rheumatoid arthritis. Egypt Rheumatol. 2014;36(1):51–6.

[28] Wang YH, Sun HY, Liu YQ, Gong XY, Xu Y, Zong QQ, et al. Health-related quality of life in Chinese SLE patients: evidence from 1568 SLE patients and 2610 healthy controls. Qual Life Res. 2024;33(1):207–18. 37824058 10.1007/s11136-023-03516-9

[29] WHO. Musculoskeletal conditions. WHO Fact Sheets. 2021. Available from: https://www.who.int/news-room/fact-sheets/detail/musculoskeletal-conditions

[30] Rosa-Gonçalves D, Bernardes M, Costa L. Quality of life and functional capacity in patients with rheumatoid arthritis – Cross-sectional study. Reumatol Clin. 2018;14(6):314–8. 28400099 10.1016/j.reuma.2017.03.002

[31] Awada S, Ajrouche R, Shoker M, Al-Hajje A, Rachidi S, Zein S, et al. Rheumatoid arthritis in the Lebanese adults: impact on health-related quality of life. J Epidemiol Glob Health. 2019;9(4):281–7. 31854170 10.2991/jegh.k.190820.001 PMC7310793

[32] Wang Y, Zhao Y, Feng X, Tian X. The impact of systemic lupus erythematosus on health-related quality of life assessed using the SF-36: A systematic review and meta-analysis. Psychol Health Med. 2019;24(9):978–91. 30943791 10.1080/13548506.2019.1587479

[33] Kaya T, Mutlu EK, Gunaydin R, Goksel Karatepe A, Sivas F. Assessment of health-related quality of life in patients with ankylosing spondylitis: A cross-sectional study using SF-36. Med (Kaunas). 2023;59(9):1613.

[34] Alkan BM, Fidan F, Tosun A, Ardıçoğlu Ö. Quality of life and self-reported disability in patients with knee osteoarthritis. Mod Rheumatol. 2014;24(1):166–71. 24261774 10.3109/14397595.2013.854046

[35] Moreira P, Pereira M, Branco J, Santos RA, Ferreira PL. Trajectories of physical function and quality of life in people with hip and knee osteoarthritis: a 10-year longitudinal study. BMC Public Health. 2023;23(1):1723. 37480019 10.1186/s12889-023-16167-9 PMC10362599

[36] Hung HM, Chen MF, Chen CH. Impacts of fatigue, stress, and perceived health status on women with rheumatic diseases: A comparison study. J Nurs Res. 2020;28(3):e116. 31688342 10.1097/JNR.0000000000000354

[37] Jafari F, Mobini M, Moradi S, Dashti Dargahloo S, Ghafour I, Elyasi F. Health-related quality of life and related factors in patients with fibromyalgia: A cross-sectional study. Iran J Psychiatry Behav Sci. 2022;16(1):e115602.

[38] Mas AJ, Carmona L, Valverde M, Ribas B, EPISER Study Group. Prevalence and impact of fibromyalgia on function and quality of life in individuals from the general population: results from a nationwide study in Spain. Clin Exp Rheumatol. 2008;26(4):519–26. 18799079

[39] Turkyilmaz AK, Kurt EE, Karkucak M, Capkin E. Sociodemographic characteristics, clinical signs and quality of life in patients with fibromyalgia. Eurasian J Med. 2012;44(2):88–93. 25610216 10.5152/eajm.2012.21 PMC4261285

[40] Soliman AM, Mohammed AE, Khaled SM, Ahmed SE. Prevalence of fibromyalgia among university students and its impact on health-related quality of life: a cross-sectional study. BMC Public Health. 2023;23(1):1329. 38057749 10.1186/s12889-023-17329-5 PMC10702101

[41] Wolfe F, Michaud K, Li T, Katz RS. EQ-5D and SF-36 quality of life measures in systemic lupus erythematosus: comparisons with rheumatoid arthritis, noninflammatory rheumatic disorders, and fibromyalgia. J Rheumatol. 2010;37(2):296–304. 20032098 10.3899/jrheum.090778

[42] Tander B, Cengiz K, Alayli G, Ilhanli I, Canbaz S, Canturk F. A comparative evaluation of health-related quality of life and depression in patients with fibromyalgia syndrome and rheumatoid arthritis. Rheumatol Int. 2008;28(8):859–65. 18317770 10.1007/s00296-008-0551-6

[43] Salaffi F, Sarzi-Puttini P, Girolimetti R, Atzeni F, Gasparini S, Grassi W. Health-related quality of life in fibromyalgia patients: a comparison with rheumatoid arthritis patients and the general population using the SF-36 health survey. Clin Exp Rheumatol. 2009;27(5 Suppl 56):S67–74. 20074443

[44] Galvez-Sánchez CM, Duschek S, Reyes del Paso GA. Is reduced health-related quality of life a primary manifestation of fibromyalgia? A comparative study with rheumatoid arthritis. Psychol Health. 2024;39(4):517–35. 10.1080/08870446.2022.2085705 35694814

[45] Smolen JS, Aletaha D, McInnes IB. Rheumatoid arthritis. Lancet. 2016;388(10055):2023–38. 27156434 10.1016/S0140-6736(16)30173-8

[46] Feng J, Yu L, Fang Y, Zhang X, Li S, Dou L. Correlation between disease activity and patient-reported health-related quality of life in rheumatoid arthritis: A cross-sectional study. BMJ Open. 2024;14:e082020. 38697757 10.1136/bmjopen-2023-082020 PMC11086289

[47] Boussaid S, Jeriri S, Rekik S, Hannech E, Jammali S, Cheour E, et al. Influencing factors in Tunisian rheumatoid arthritis patients’ quality of life: burden and solutions. Curr Rheumatol Rev. 2023;19(3):314–20. 36411572 10.2174/1573397119666221118143624

[48] Bretterklieber A, Painsi C, Avian A, Wutte N, Aberer E. Impaired quality of life in patients with systemic sclerosis compared to the general population and chronic dermatoses. BMC Res Notes. 2014;7:594. 25183055 10.1186/1756-0500-7-594 PMC4175285

[49] Canpolat Ö, Yurtsever S. The quality of life in patients with Behçet’s disease. Asian Nurs Res (Korean Soc Nurs Sci). 2011;5(4):229–35. 25030525 10.1016/j.anr.2011.12.003

[50] Cagnie B, Coppieters I, Denecker S, Six J, Danneels L, Meeus M. Central sensitization in fibromyalgia? A systematic review on structural and functional brain MRI. Semin Arthritis Rheum. 2014;44(1):68–75. 24508406 10.1016/j.semarthrit.2014.01.001

[51] Nelson AE, Allen KD, Golightly YM, Goode AP, Jordan JM. A systematic review of recommendations and guidelines for the management of osteoarthritis. Semin Arthritis Rheum. 2014;43(6):701–12. 24387819 10.1016/j.semarthrit.2013.11.012

[52] Broderick CR, McIntosh AS, Pengel L, Jan S, Heller GZ. Physical activity and sleep in people with arthritis: a cross-sectional comparison of rheumatoid arthritis and osteoarthritis. BMC Public Health. 2021;21(1):2157.34819057

[53] Jansen JP, Buckley F, Dejonckheere F, Ogale S. Comparative efficacy of biologics as monotherapy and in combination with methotrexate on patient reported outcomes in rheumatoid arthritis: A systematic review and network meta-analysis. Health Qual Life Outcomes. 2014;12:102. 24988902 10.1186/1477-7525-12-102 PMC4101713

[54] Bekarissova S, Bekarisov O, Bekaryssova D. An integrated approach to the treatment of rheumatic diseases: the role of psychological interventions. Rheumatol Int. 2024;44(12):2727–35. 39400563 10.1007/s00296-024-05728-9

[55] Bannuru RR, Osani MC, Vaysbrot EE, Arden NK, Bennell K, Bierma-Zeinstra SMA, et al. OARSI guidelines for the non-surgical management of knee, hip, and polyarticular osteoarthritis. Osteoarthritis Cartilage. 2019;27(11):1578–89. 31278997 10.1016/j.joca.2019.06.011

[56] Salaffi F, Carotti M, Gasparini S, Intorcia M, Grassi W. Health-related quality of life in patients with rheumatoid arthritis: evaluation by the SF-36 instrument. Reumatismo. 2009;61(2):105–15.

[57] Aletaha D, Smolen JS. Diagnosis and management of rheumatoid arthritis: A review. JAMA. 2018;320(13):1360–73. 30285183 10.1001/jama.2018.13103

[58] Heckert SL, Maassen JM, Nevins I, Baudoin P, Steup-Beekman GM, Huizinga TWJ et al. Long-term clinical outcomes in early rheumatoid arthritis that was treated-to-target in the best and IMPROVED studies. Rheumatology (Oxford). 2024. 10.1093/rheumatology/keae212 10.1093/rheumatology/keae212 PMC11879312 38561181

[59] Emery P, Hammoudeh M, FitzGerald O, Combe B, Martin-Mola E, Buch MH, et al. Sustained remission with etanercept tapering in early rheumatoid arthritis. N Engl J Med. 2014;371(19):1781–92. 25372086 10.1056/NEJMoa1316133

[60] Fonseca JE, Canhão H, Teixeira da Costa JC, Pereira da Silva JA, Viana Queiroz M. Global functional status in rheumatoid arthritis: disease duration and patient age. Clin Rheumatol. 2002;21:32–4. 11954881 10.1007/s100670200008

[61] Bertsias A, Flouri ID, Repa A, Avgoustidis N, Kalogiannaki E, Pitsigavdaki S, et al. Patterns of comorbidities differentially affect long-term functional evolution and disease activity in patients with ‘difficult to treat’ rheumatoid arthritis. RMD Open. 2024;10(1):e003808. 38242549 10.1136/rmdopen-2023-003808 PMC10806522

