# Supplementary material for: Health-related quality of life among people with rheumatic and musculoskeletal diseases in Cyprus: a cross-sectional study of disease burden and time since diagnosis
Source: BMC Rheumatol. 2026 Jan 20;10:14. doi: 10.1186/s41927-026-00617-z (PMC12905859; doi:10.1186/s41927-026-00617-z)
Supplement: Supplementary file 1 — Supplementary Material 1 [file 41927_2026_617_MOESM1_ESM.docx]

| **Table 1: Comparison of clinic-recruited and online- recruited participants** | | | |
| --- | --- | --- | --- |
| **Variable** | **Online (n=316)** | **Clinical (n=473)** | **p-value** |
| SF-12 PCS | 37.67 ± 19.80 | 37.61 ± 20.61 | 0.966 |
| SF-12 MCS | 46.11 ± 19.77 | 46.49 ± 20.84 | 0.799 |

*Values are presented as mean ± standard deviation. p-values were derived from independent samples t-tests.*
